# Variations in the frequencies of polymorphisms in the *CYP2C9* gene in six major ethnicities of Pakistan

**DOI:** 10.1038/s41598-020-76366-x

**Published:** 2020-11-09

**Authors:** Sagheer Ahmed, Nadeem Altaf, Mahnoor Ejaz, Aisha Altaf, Aneela Amin, Kholood Janjua, Arif Ullah Khan, Imran Imran, Saeed Khan

**Affiliations:** 1 Department of Basic Medical Sciences, Shifa College of Pharmaceutical Sciences, Shifa Tameer-E-Millat University, Sector H-8, Islamabad, 44000 Pakistan; 2 Overseas Pakistanis Foundation Boys College, Sector H-8, Islamabad, Pakistan; 3 Shifa Clinical Research Center, Shifa International Hospital, Islamabad, Pakistan; 4 Riphah Institute of Pharmaceutical Sciences, Riphah International University, Islamabad, Pakistan; 5 Department of Pharmacology, Faculty of Pharmacy, Bahauddin Zakariya University, Multan, Pakistan; 6 Dow International Medical College, Dow University of Health Sciences, Karachi, Pakistan

**Keywords:** Genetics, Molecular biology

## Abstract

Genetic variation in cytochrome P450 (*CYP) 2C9* is known to cause significant inter-individual differences in drug response and adverse effects. The frequencies of CYP2C9*2 and CYP2C9*3, both of which are responsible for the low activity of the enzyme, are not known in the Pakistani population. Therefore, we screened various ethnic groups residing in Pakistan for these polymorphisms. A total of 467 healthy human volunteers were recruited from six major ethnicities of Pakistan after written informed consent. Our results indicate that about 20% of the Pakistani population has a genotype containing at least one low activity allele. Ethnic Punjabi and Pathan populations had the highest frequencies of wild type genotypes while Urdu, Seraiki, and Sindhi populations showed higher rates of both low activity genotypes. The Baloch population showed the highest rates of low activity genotypes with less than 50% of the samples showing wild type genotypes, suggesting that more than half of the Baloch population possesses low activity genotypes. The frequencies found in various ethnic groups in Pakistan were comparable with ethnicities in the South Asian region except for the Baloch population. These results suggest that pharmacogenetics screening for low activity genotypes may be a helpful tool for clinicians while prescribing medications metabolized by CYP2C9.

## Introduction

Genetic polymorphisms in cytochrome P450(CYP) genes can lead to altered enzymatic activity with consequent variation in the pharmacokinetics of a metabolized drug. This variation in the pharmacokinetics of the drug may lead to a change in the response of the drug. It is usually the result of single nucleotide polymorphisms (SNPs) or copy number variations in CYP genes. Therefore, these genetic variants play a crucial role in determining drug pharmacokinetics, non-responder phenomenon, and toxicity^1^. In recent years, these CYP polymorphisms have become important pharmacogenetic biomarkers for drug dosing, efficacy, and safety. However, there is considerable variation in the distribution of CYP alleles in different populations around the world, which may result in variation in drug response and has important implications for personalized treatment.

One of the most important subfamilies of CYP is CYP2C, which consists of four enzymes: CYP2C8, CYP2C9, CYP2C18, and CYP2C19. Of these, the enzyme expressed most abundantly is CYP2C9, which is significantly involved in the metabolism of many important clinical drugs. Based on quantification by mass spectrometry, CYP2C9 is about 20% of the total hepatic P450 protein content^2^. Another important site of expression is the gastrointestinal tract^3^. CYP2C9 is responsible for the metabolism of approximately 13% of the drugs metabolized by the cytochrome-catalyzed reactions^4^. Many of these drugs are clinically very important but have a narrow therapeutic index (e.g., warfarin). Therefore, inter-individual variation in CYP2C9 expression and activity may result in inter-individual variation in drug response and safety. Genetic polymorphisms in *CYP2C9* and drug-drug interactions further increase the variation in drug response^1,5,6^.

*CYP2C9* gene contains several variants, but two of the most prevalent, investigated, and influential of them are rs1799853 and rs1057910, also known as CYP2C9*2 and CYP2C9*3 polymorphisms, respectively. CYP2C9*2 results in an amino acid substitution from Arg to Cys as a consequence of C > T transition in the *CYP2C9* gene at position 430 (c.430C > T, p.Arg144Cys). CYP2C9*3 causes an amino acid substitution from Ilu to Leu as a result of a transversion, A > T in the *CYP2C9* gene (c.1075A > C, p.Ile359Leu)^7^. The activity of CYP2C9 is significantly reduced as a result of these polymorphisms, and in the Caucasian population, they are responsible for a majority of decreased CYP2C9 activity phenotypes^7^. Both of these variations decrease the rate of phenytoin hydroxylation^8^. Hydroxylation of S-warfarin is impaired by CYP2C9*2^6^, while the metabolism of tolbutamide is decreased by CYP2C9*3^9^. Benzo[a]pyrene, an important lung carcinogen, is also metabolized by CYP2C9, and therefore, SNPs in this gene also carry the risk of lung cancer^10^. Therefore, these SNPs not only affect drug response and adverse effects, but they are also associated with certain disease phenotypes.

Interpopulation differences in drug responses are well known, and in some cases, they correspond to differences in the frequency of associated genetic markers, especially CYP genes. That is why differences in *CYP2C9* allele distribution have been described for various populations. Pakistan is a culturally diverse country, but little is known about the distribution of *CYP2C9* genetic polymorphism in this country of over 200 million people. Therefore, we intended to determine the frequencies of these polymorphisms in the Pakistani population, with samples drawn from six of its most populous ethnic groups. We specifically investigated the samples of various ethnic populations from Pakistan to examine the frequencies of CYP2C9*1, CYP2C9*2, and CYP2C9*3 and then compared them with previous findings in other populations.

## Results

### Allelic frequencies

The allelic frequencies of CYP2C9*1, CYP2C9*2, and CYP2C9*3 in the Pakistani population were found to be 0.876, 0.059, and 0.064, respectively (Table 1), while for the ethnic Punjabi population, the frequencies were 0.951, 0.034, and 0.015 respectively. In the ethnic Pathan population, the observed frequencies were in a similar range (0.943, 0.032, 0.032 for CYP2C9*1, CYP2C9*2, and CYP2C9*3, respectively). In Urdu speaking population, the rate of CYP2C9*1, CYP2C9*2, and CYP2C9*3 were slightly different at 0.820, 0.078, and 0.023, respectively. The Seraiki population was found to have CYP2C9*1, CYP2C9*2, and CYP2C9*3 at frequencies of 0.882, 0.46, and 0.73, respectively. The results obtained from the Baloch population diverged significantly. In the Baloch population, the frequencies of CYP2C9*1, CYP2C9*2, and CYP2C9*3 were found to be 0.620, 0.160, and 0.220, respectively, whereas, in the Sindhi population, the frequencies were 0.830, 0.090, and 0.080 respectively (Table 1).

**Table 1:** Allelic frequencies of various ethnic groups in Pakistan.

Ethnic group	n	*1 (95% CI)	*2 (95% CI)	*3 (95% CI)
Pakistan	467	0.876 (84.61–90.59)	0.059 (3.76–8.04)	0.064 (4.18–8.62)
Punjabi	169	0.951 (91.85–98.35)	0.034 (0.67–6.13)	0.015 (− 0.33–3.33)
Pathan	79	0.943 (89.19–99.41)	0.032 (− 0.68–7.08)	0.025 (− 0.94–5.94)
Urdu	64	0.820 (72.59–91.41)	0.064 (1.14–12.25)	0.101 (5.67–19.39)
Seraiki	55	0.882 (79.69–96.73)	0.046 (− 0.94–10.14)	0.073 (0.43–14.17)
Balochi	50	0.620 (48.55–75.45)	0.160 (5.84–26.16)	0.220 (10.52–33.48)
Sindhi	50	0.830 (72.59–93.41)	0.090 (1.07–16.93)	0.080 (0.48–15.51)

### Genotype frequencies

The observed genotype frequencies were 80.3% for CYP2C9*1*1, 6.2% for CYP2C9*1*2, 8.4% for CYP2C9*1*3, 0.6% for CYP2C9*2*2 and 4.5% for CYP2C9*2*3 in Pakistani population (Table 2). No one was found to be homozygous for CYP2C9*3*3. Genotype frequencies in Punjabi, Pathan, Urdu, Seraiki, Baloch, and Sindhi populations are shown in Table 2. In the Punjabi population, all the genotype frequencies except CYP2C9*3*3 were present, whereas, in the Pathan population, no CYP2C9*2*3 and CYP2C9*3*3 genotypes were observed. While the Pathan population lacked CYP2C9*2*3 and CYP2C9*3*3 genotypes, Urdu and Seraiki speaking populations were deficient in CYP2C9*2*2 and CYP2C9*3*3 genotypes. The genotype frequencies of the Baloch population were significantly different from other ethnic groups. In the Baloch population, the frequencies of CYP2C9*1*1 were comparatively low at 46%, and decreased activity genotypes were considerably higher. Like Urdu and Seraiki populations, the Baloch population also lacked CYP2C9*2*2 and CYP2C9*3*3 genotypes. This was also true for the Sindhi population in which the frequencies of CYP2C9*1*1 were comparable to the Urdu speaking population (Table 2).

**Table 2:** Genotype frequencies of various ethnic groups in Pakistan.

Genotype	n	Observed genotype frequency (CI)	Expected genotype frequency by HW law
**Pakistani**			
*1*1	375	80.3 (76.7–83.9)	79.85
*1*2	29	6.2 (4.02–8.38)	7.08
*1*3	39	8.4 (5.89–10.91)	8.00
*2*2	3	0.6 (0.1–1.3)	0.16
*2*3	21	4.5 (2.62–6.38)	4.91
**Punjab**i			
*1*1	155	91.7 (87.54–95.86)	91.75
*1*2	8	4.7 (1.51–7.89)	4.58
*1*3	4	2.4 (0.09–4.71)	2.36
*2*2	1	0.6 (0.0–1.76)	0.05
*2*3	1	0.6 (0.0–1.76)	1.24
**Pathan**			
*1*1	72	91.1 (84.48–97.72)	88.70
*1*2	1	1.3 (0.0–3.93)	6.00
*1*3	4	5.1 (0.0–10.22)	4.96
*2*2	2	2.5 (0.0–6.13)	0.10
*2*3	0	0.0	0.24
Urdu			
*1*1	46	71.9 (60.89–82.91)	72.0
*1*2	5	7.8 (1.23–14.37)	7.41
*1*3	8	12.5 (4.4–20.6)	11.57
*2*2	0	0.0	0.19
*2*3	5	7.8 (1.23–14.37)	6.83
**Seraiki**			
*1*1	44	80.0 (69.43–90.57)	80.00
*1*2	3	5.5 (− 0.53–11.53)	5.32
*1*3	6	10.9 (2.66–19.14)	10.24
*2*2	0	0.0	0.08
*2*3	2	3.6 (− 1.32–8.52)	4.36
**Balochi**			
*1*1	23	46.0 (32.19–59.81)	46.44
*1*2	5	10.0 (1.68–18.32)	9.10
*1*3	11	22.0 (10.52–33.48)	18.44
*2*2	0	0.0	0.40
*2*3	11	22.0 (10.52–33.48)	25.62
**Sindhi**			
*1*1	35	70.0 (57.3–82.7)	70.58
*1*2	7	14.0 (4.38–23.62)	12.83
*1*3	6	12.0 (2.99–21.01)	11.12
*2*2	0	0.0	0.58
*2*3	2	4.0 (− 1.43–9.43)	4.89

### Comparison with worldwide populations

Comparison with the worldwide and regional populations revealed significant differences in the frequencies of CYP2C9*2. Colombian, Puerto Rican, Spanish, and Italian populations showed significantly higher frequencies while Han Chinese, Bengali, and Indian Telugu population displayed a significantly low frequency of CYP2C9*2. Frequencies of this allele in Mexican, Peruvian, Finnish, British, Gujrati Indian, and Sri Lankan Tamil populations were not statistically different from Pakistani frequencies (Table 3). Allelic frequencies of CYP2C9*3 in Peruvian and Chinese Dai populations were found lower than the Pakistani population, while Bengali and Gujrati Indian populations showed significantly higher frequencies (Table 4). CYP2C9*3 frequencies in Colombian, Mexican, Puerto Rican, Han & Southern Han Chinese, Japanese, Vietnamese, Finnish, British, Spanish, Italian, Sri Lankan Tamil, and Indian Telgu populations were not statistically different from Pakistani frequencies observed in our investigation (Table 4). Frequencies of CYP2C9*2 and CYP2C9*3 frequencies in Pakistanis from Lahore were also in agreement with our study (Table 3 & 4).

**Table 3:** Comparison of CYP2C9*2 allelic frequencies observed in Pakistan with other populations.

Population	CYP2C9*1	CYP2C9*2	Chi-square statistic	p-value
CLM	165	23	9.30	0.002285
MXL	115	13	3.20	0.073297
PEL	166	4	3.71	0.053976
PUR	179	29	15.59	0.000079
CHS	209	1	11.03	0.000895
CEU	168	30	19.50	0.000010
FIN	182	16	1.19	0.274819
GBR	166	16	2.71	0.099223
IBS	184	30	16.17	0.000058
TSI	181	33	21.62	0.000010
BEB	169	3	5.19	0.022599
GIH	196	10	0.40	0.525495
ITU	199	5	4.14	0.041707
PJL	182	10	0.17	0.72285
STU	198	6	3.03	0.081610

CLM-Colombian in Medellin, Colombia, MXL-Mexican ancestory in Los Angeles, California, PEL-Peruvian in Lima, Peru, PUR-Puerto Rican in Puerto Rico, CHS-Southern Han Chinese, China, CEU-Utah residents with northern and western European ancestory, FIN-Finnish in Finland, GBR-British in England and Scotland, IBS-Iberian population in Spain, TSI-Toscani in Italy, BEB-Bengali in Bangladesh, GIH-Gujrati Indian in Houston, TX, ITU-Indian Telgu in UK, PJL-Punjabi in Lahore, Pakistan, STU-Sri Lankan Tamil in the UK.

**Table 4:** Comparison of CYP2C9*3 allelic frequencies observed in Pakistan with other populations.

Population	CYP2C9*1	CYP2C9*3	Chi-square statistic	p-value
CLM	176	12	0.00	0.983300
MXL	125	3	3.35	0.066859
PEL	168	2	7.47	0.006268
PUR	199	9	1.31	0.250987
CDX	181	5	3.95	0.046597
CHB	198	8	1.94	0.163451
CHS	200	10	0.82	0.363867
JPT	204	4	6.51	0.10700
KHV	191	7	2.44	0.117672
CEU	185	13	0.00	0.941230
FIN	187	11	0.20	0.647096
GBR	169	13	0.12	0.719721
IBS	196	18	1.08	0.297420
TSI	196	18	1.08	0.297420
BEB	152	20	5.86	0.015470
GIH	179	27	10.69	0.001076
ITU	183	21	3.79	0.051460
PJL	173	19	2.94	0.086270
STU	184	20	2.92	0.087132

CLM-Colombian in Medellin, Colombia, MXL-Mexican ancestory in Los Angeles, California, PEL-Peruvian in Lima, Peru, PUR-Puerto Rican in Puerto Rico, CDX-Chinese Dai in Xishuangbanna, China CHB-Han Chinese in Beijing, China, CHS-Southern Han Chinese, China, JPT-Japanese in Tokyo, Japan, KHV-Kinh in Ho Chi Minh City, Vietnam, CEU-Utah residents with northern and western European ancestory, FIN-Finnish in Finland, GBR-British in England and Scotland, IBS-Iberian population in Spain, TSI-Toscani in Italy, BEB-Bengali in Bangladesh, GIH-Gujrati Indian in Houston, TX, ITU-Indian Telgu in UK, PJL-Punjabi in Lahore, Pakistan, STU-Sri Lankan Tamil in the UK.

## Discussion

**Figure 1:**
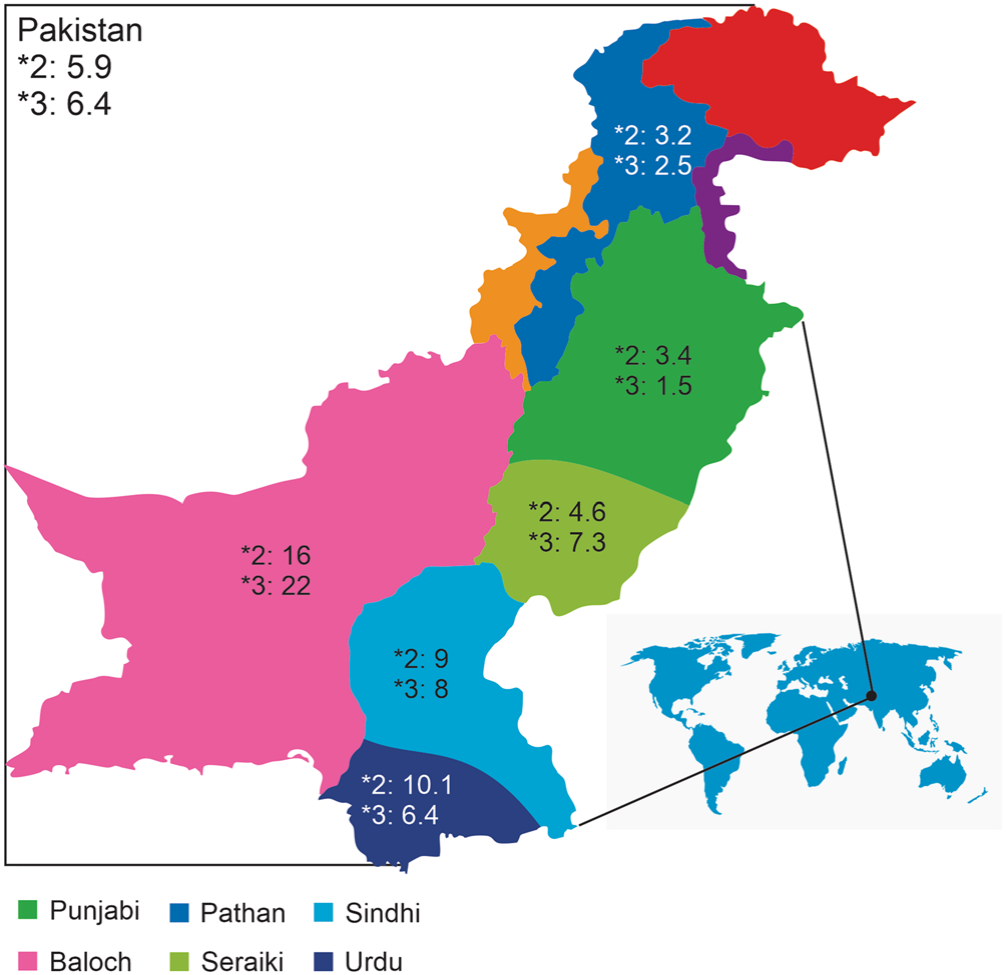
Geographical representation of the ethnic groups investigated in the study. The figure was created using CorelDRAW Graphic Suite 2020 **(**https://www.coreldraw.com/en/).

Pakistan is one of the most populous countries in the world, with an estimated population of over 220 million people. Pakistan boasts a relatively young population that comes from diverse cultural and ethnic backgrounds. Despite being home to one of the biggest populations in the world, studies investigating genetic variations responsible for drug response are scarce. There are several dozen ethnic groups in Pakistan. However, the six ethnicities we selected for our study represent more than 94% of the Pakistani population. The biggest ethnic group in Pakistan are Punjabis, followed by Pathan, Sindhi, Saraiki, Urdu, and Baloch ethnic groups. A geographical map indicating the regions where selected ethnicities primarily reside and the distribution CYP2C9 genetic frequencies in those ethnicities are shown in Fig. 1.

The allelic frequencies of CYP2C9*2 and CYP2C9*3 observed in the present study were found to agree with previously reported frequencies around the world (Table 3 & 4). The frequency of CYP2C9*1 in Pakistan was closest to the one found in America. However, the frequency of CYP2C9*2 was higher in the American population, and CYP2C9*3 was slightly higher in the Pakistani population ^11^. In South Asia, Bangladesh was found to have a similar frequency of CYP2C9*1, but the frequency of CYP2C9*2 in Bangladesh was significantly low, and that of CYP2C9*3 was significantly high compared to what was observed in Pakistani population ^12^ (Table 3 & 4).

Frequencies of CYP2C9*2 were relatively high in the Pakistani population compared to many Asian populations such as Japanese, Korean, Chinese Taiwanese, in which this allele was absent^7,13,14^. Many regional populations such as Indians and Sri Lankans did show significant differences in frequencies of CYP2C9*2 allele ^15–18^. However, the Pakistani population displayed slightly lower frequencies of this allele compared to regional populations such as in Bengali and Gujrati Indians. Among the European populations, Swedish, Turkish^19–21^, Spanish and Italian populations had higher frequencies (Table 3) while Finnish and British populations, while displaying higher frequencies, were not statistically different from Pakistani population. The frequencies of the CYP2C9*3 allele in the Pakistani population were found to be similar to many European populations, including British, Finnish, Spanish, and Italian (Table 4). However, Peruvian and Chinese Dai populations showed statistically lower frequencies. Frequencies of CYP2C9*3 found in some regional population such as Bengalis and Gujrati Indians, were higher while in others, such as in Indian Telugu, and Sri Lankan Tamil were in agreement with our results (Tables 3, 4).

Among different ethnicities, Punjabi and Pathan populations had the highest frequencies of the CYP2C9*1 allele, while the CYP2C9*2 allele frequencies were also in a similar range. However, CYP2C9*3 frequencies were different between these two ethnicities, with the Pathan population showing much greater frequencies compared to the Punjabi population. Urdu and Seraiki populations had slightly lower frequencies of the wild type allele compared to Punjabi and Pathan populations. However, the allelic frequency of CYP2C9*2 was higher in the Urdu speaking population, while CYP2C9*3 was found higher in the Seraiki population. Baloch populations samples showed results very different from any other ethnic population. The baloch population had the lowest frequency of wild type allele, while the frequency of CYP2C9*2 was the highest among Pakistani populations. Similarly, the Baloch frequencies of CYP2C9*3 were also the highest among Pakistani ethnicities. The pattern in the Sindhi population was similar to Urdu and Seraiki populations.

While analyzing genotype frequencies, Punjabi and Pathan population samples showed similar frequencies of wild type genotype, CYP2C9*1*1. However, unlike Punjabi population samples, Pathan population samples lacked the CYP2C9*2*3 genotype (Table 2). Urdu and Seraiki population samples, although having similar frequencies of wild type allele, had different wild type genotypes. This implies that roughly 30% of the Urdu speaking population has a *CYP2C9* genotype with at least one low activity allele. This was found to be true for the Sindhi population as well in which the frequencies of CYP2C9*1*1 were reported to be 70%. In the Baloch population, wild type *CYP2C9* genotype was reported in only 46% samples, and therefore, indicates that more than half the population may possess at least one low activity allele (Table 2). This represents a significant fraction of the Baloch population with a potentially variable response and/or enhanced adverse effects when drugs metabolized by *CYP2C9* are administered.

The Pakistani population is a heterogeneous mixture of Asian, Middle Eastern, and European populations partly because of the Arab invasion of the eighth century and British invasions of the eighteenth and nineteenth centuries, and partly owing to its high geographic and ethnic diversity^22^. The genetic structure of various Pakistani populations have been analyzed and several distinct variants identified among different ethnicities by global projects such as the 1000 Genome Project and Human Genome Diversity project ^12,23^. Some studies indicate that the genetic structure of these ethnicities is closely related to both South Indian and European populations ^24^ while others suggest Pakistani ethnicities to be similar to European populations ^25,26^. The extreme differences observed in the Balochi population may be due to their diverse ancestry belonging to Aryan, Arab, Persian, Turkish, Kurdish, Dravidian, Sewais, and black African lineages ^27^.

Genetic information about patients' *CYP2C9* gene is likely to help physicians prescribe to patients the most suitable and safest drug based on their genetic make-up. With roughly 13% clinically available drugs metabolized by CYP2C9 enzyme^28^ and over 2.6 billion unit doses of drugs dispensed in Pakistan annually, the number of unit doses metabolized by the CYP2C9 enzyme in Pakistan annually is over 332 million. Our study shows that about 20% of Pakistan's population has a *CYP2C9* genotype that contains at least one low activity allele. These results indicate that over 66 million doses of drugs dispensed annually in Pakistan may not have desired effects as patients receiving these medications possess a low activity *CYP2C9* allele. In patients receiving a drug that requires activation through CYP2C9, a lack of response could be expected. On the contrary, if a drug is inactivated by CYP2C9, then increased frequency and severity of adverse effects would be a more likely outcome. With *CYP2C9* genotype information at hand, physicians will have a choice to change the drug or dose of the drug to provide maximum therapeutic benefit to the patient and/or prevent the undesired and excessive adverse effects.

To our knowledge, this is the first study to report frequencies of *CYP2C9* gene polymorphisms in various ethnicities of the Pakistani population. Although there have been a few studies from Pakistan in which frequency of CYP2C9*2 and CYP2C9*3 were reported^29–31^, all of these studies involved patients with different diseases and, therefore, unable to capture the actual frequency of these polymorphisms in a general Pakistani population. The frequencies of CYP2C9*2 and CYP2C9*3 reported in these publications are 5.1% for CYP2C9*2, 15.4% for CYP2C9*3 in breast cancer patients^29^, 4.45% for CYP2C9*2, 22.8% for CYP2C9*3 in cardiovascular patients taking warfarin^31^ and 12.1% for CYP2C9*2, 14.1% for CYP2C9*3 in heart valve replacement patients taking warfarin^30^. These frequencies vary significantly from one study to another and are also different from the ones we have reported for the healthy Pakistani population in this study. Frequencies of both low activity alleles were significantly higher in these studies than what we observed in our study. For example, frequencies of the CYP2C9*3 allele were four times higher in one of these studies and more than twice higher in the rest of the two studies. This may be because some polymorphisms are associated with certain diseases, and therefore, their frequencies in the patient groups would be different from a normal healthy population. Large differences in the sample size in these studies could also partly explain the variations observed in allelic frequencies. Another publication reporting the *CYP2C9* gene polymorphisms in the Pakistani population also had participants who were heart valve replacement patients taking warfarin^32^. However, the allelic frequencies reported in that study were in agreement with ours, although the frequencies of CYP2C9*2 were slightly on the lower side. This study was carried out in patients with Punjabi ethnicity only. Furthermore, patient samples reported in these studies were obtained from a single geographical location and, therefore, may not represent entire Pakistan, which is a large country with a population of over 220 million people having varied ethnic backgrounds.

In conclusion, both the CYP2C9*2 and CYP2C9*3 allelic variants are found in the Pakistani population, and CYP2C9*3 was slightly more common than CYP2C9*2. One limitation of our study is we were unable to find the true CYP2C9*1 allele due to the genotyping method we employed in our research study. Individuals were genotyped CYP2C9*1 when neither CYP2C9*2 nor CYP2C9*3 was detected. Most of the polymorphisms demonstrated in our study were heterozygous. No CYP2C9*3*3 homozygosity was seen in our study, and only 3 (less than 1%) were homozygous for CYP2C9*2*2. This suggests that the homozygous polymorphism is rare in the Pakistani population. The frequency of these polymorphisms was found to be slightly different in different ethnic populations in Pakistan except for Baloch population samples, which showed an unusually high frequency of these polymorphisms. We recommend that genotyping of the *CYP2C9* gene in patients on drugs such as warfarin, phenytoin, etc., may help to overcome the drug toxicity, chose the right alternative, and guide in therapeutic drug monitoring.

## Methods

The study was approved by the Institutional Review Board and Ethics Committee of Shifa Tameer-e-Millat University, Islamabad, Pakistan, through approval number IRB#990–265-2018. Informed written consent was obtained from all participating individuals. All experiments were performed in accordance with relevant guidelines and regulations. A total of 467 unrelated individuals from a healthy population were recruited for the present study. The study cohort consists of six major ethnicities of Pakistan, including Punjabis, Pathan, Sindhi, Balochi, Seraiki, and Urdu speaking. Ethnicity was self-reported. Five milliliters of venous blood was drawn into a sterile tube containing EDTA as an anti-coagulant and were stored at 4^ο^C. Genomic DNA was isolated using Gene Jet Genomic DNA extraction Kit (ThermoScientific) and was quantified using 1% agarose gel electrophoresis. Isolated genomic DNA was stored at – 20 °C until further processing^33^.

### Genotyping

CYP2C9*2 and *3 were genotyped using ARMS-PCR (Allele Refractory Mutation System- Polymerase Chain Reaction) using a pair of outer primers and a pair of inner primers as described previously ^34^. PCR for both the SNPs was performed in a single tube with a total reaction volume of 25 µl containing 12.5 µl of 2X Dream Taq Master mix (ThermoScientific), 0.5 pM of 2C9*2 wild type reverse primer, 1.5 pM of 2C9*2 mutant reverse primer, 3.0 pM of common forward primer, 1.0 pM of 2C9*3 wild type forward primer, 2.0 pM of 2C9*3 mutant forward primer, 3.0 pM of common reverse primer and 3 µl of template DNA (20–50 ng/μl). Thermal profile was as follows: initial denaturation at 95^ο^C for 10 min followed by 37 cycles with denaturation at 95^ο^C for 45 s, 45 s of primer annealing at 58^ο^C, initial extension at 72 ^ο^C for 45 s, and a final extension at 72 ^ο^C for 7 min. For visualization, 12 µl of PCR product was directly loaded onto 4% agarose gel. The PCR products for 2C9*2 had 105 bp fragment for the wild type allele and 114 bp fragment for the mutant allele, whereas 2C9*3 had 159 bp fragment for the wild type allele and 168 bp fragment for the mutant allele. Individuals were genotypes 2C9*1 when neither 2C9*2 nor 2C9*3 was detected. More than one-quarter of the total samples were sent for sequencing to validate further the results obtained through ARMS-PCR.

### Statistical analysis

Allelic Data were compiled according to the genotype and allele frequencies estimated from the observed numbers of each specific allele. The frequency of each allele and genotype in our samples is given together with the 95% confidence interval. The confidence interval for proportions was calculated using the formula (CI = p ± (1.96 × SE), SE = qrt [ p(1—p) / n ], p = proportion, n = sample size). Chi-squared test and p values were calculated using observed and expected frequencies as per the Hardy–Weinberg equation.

### Ethical statement

The study was approved by the Institutional Review Board and Ethics Committee of the Shifa International Hospital and Shifa Tameer-e-Millat University, Islamabad, Pakistan.

## References

[1] Zhou S-F, Zhou Z-W, Yang L-P, Cai J-P (2009). Substrates, inducers, inhibitors, and structure-activity relationships of human Cytochrome P450 2C9 and implications in drug development. Curr. Med. Chem..

[2] Zhang H-F (2016). Physiological content and intrinsic activities of 10 cytochrome P450 isoforms in human normal liver microsomes. J. Pharmacol. Exp. Ther..

[3] Paine MF (2006). The human intestinal cytochrome P450 ‘pie’. Drug Metab. Dispos. Biol. Fate Chem..

[4] Isvoran A (2017). Pharmacogenomics of the cytochrome P450 2C family: impacts of amino acid variations on drug metabolism. Drug Discov. Today.

[5] Zhou S-F, Zhou Z-W, Huang M (2010). Polymorphisms of human cytochrome P450 2C9 and the functional relevance. Toxicology.

[6] Rettie AE, Jones JP (2005). Clinical and toxicological relevance of CYP2C9: drug–drug interactions and pharmacogenetics. Annu. Rev. Pharmacol. Toxicol..

[7] Yoon Y-R (2001). Frequency of cytochrome P450 2C9 mutant alleles in a Korean population. Br. J. Clin. Pharmacol..

[8] Veronese ME (1993). Site-directed mutation studies of human liver cytochrome P-450 isoenzymes in the CYP2C subfamily. Biochem. J..

[9] Relling MV, Aoyama T, Gonzalez FJ, Meyer UA (1990). Tolbutamide and mephenytoin hydroxylation by human cytochrome P450s in the CYP2C subfamily. J. Pharmacol. Exp. Ther..

[10] London SJ, Daly AK, Leathart JB, Navidi WC, Idle JR (1996). Lung cancer risk in relation to the CYP2C9*1/CYP2C9*2 genetic polymorphism among African-Americans and Caucasians in Los Angeles County, California. Pharmacogenetics.

[11] Sullivan-Klose TH (1996). The role of the CYP2C9-Leu359 allelic variant in the tolbutamide polymorphism. Pharmacogenetics.

[12] Auton A (2015). A global reference for human genetic variation. Nature.

[13] Th S-K (1996). The role of the CYP2C9-Leu359 allelic variant in the tolbutamide polymorphism. Pharmacogenetics.

[14] Nasu K, Kubota T, Ishizaki T (1997). Genetic analysis of CYP2C9 polymorphism in a Japanese population. Pharmacogenetics.

[15] Adithan C (2003). Allele and genotype frequency of CYP2C9 in Tamilnadu population. Eur. J. Clin. Pharmacol..

[16] Chaudhary N (2016). Frequencies of CYP2C9 polymorphisms in North Indian population and their association with drug levels in children on phenytoin monotherapy. BMC Pediatr..

[17] Jose R (2005). CYP2C9 and CYP2C19 genetic polymorphisms: frequencies in the south Indian population. Fundam. Clin. Pharmacol..

[18] 1000 Genomes | A Deep Catalog of Human Genetic Variation. https://www.internationalgenome.org/.

[19] Stubbins MJ, Harries LW, Smith G, Tarbit MH, Wolf CR (1996). Genetic analysis of the human cytochrome P450 CYP2C9 locus. Pharmacogenetics.

[20] Yasar U (1999). Validation of methods for CYP2C9 genotyping: frequencies of mutant alleles in a Swedish population. Biochem. Biophys. Res. Commun..

[21] Sükrü Aynacioglu A (1999). Frequency of cytochrome P450 CYP2C9 variants in a Turkish population and functional relevance for phenytoin. Br. J. Clin. Pharmacol..

[22] Bhatti S, Aslamkhan M, Attimonelli M, Abbas S, Aydın HH (2017). Mitochondrial DNA variation in the Sindh population of Pakistan. Austral. J. Forensic Sci..

[23] Bergström A (2020). Insights into human genetic variation and population history from 929 diverse genomes. Science.

[24] Metspalu M (2011). Shared and unique components of human population structure and genome-wide signals of positive selection in South Asia. Am. J. Hum. Genet..

[25] Ayub Q (2003). Reconstruction of human evolutionary tree using polymorphic autosomal microsatellites. Am. J. Phys. Anthropol..

[26] Mansoor A (2004). Investigation of the Greek ancestry of populations from northern Pakistan. Hum. Genet..

[27] Ahmed, M. & Khan, G. The history of Baloch and Balochistan: a critical appraisal. 14.

[28] Zanger UM, Schwab M (2013). Cytochrome P450 enzymes in drug metabolism: regulation of gene expression, enzyme activities, and impact of genetic variation. Pharmacol. Ther..

[29] Afsar NA (2010). Genotype frequencies of selected drug metabolizing enzymes and ABC drug transporters among breast cancer patients on FAC chemotherapy. Basic Clin. Pharmacol. Toxicol..

[30] Yasmeen F (2015). Analysis of CYP2C9 polymorphisms (*2 and *3) in warfarin therapy patients in Pakistan. Association of CYP2C9 polymorphisms (*2 and*3) with warfarin dose, age. PT and INR. J. Thromb. Thrombolysis.

[31] Qayyum A (2017). Frequency of common CYP2C9 polymorphisms and their impact on warfarin dose requirement in Pakistani population. Clin. Appl. Thromb. Off. J. Int. Acad. Clin. Appl. Thromb..

[32] Siddiqi A, Khan DA, Khan FA, Naveed AK (2010). Impact of CYP2C9 genetic polymorphism on warfarin dose requirements in Pakistani population. Pak. J. Pharm. Sci..

[33] Ahmed S (2020). Genetic variations in drug-metabolizing enzyme CYP2C9 among major ethnic groups of Pakistani population. Gene.

[34] Funk M (2004). CYP2C9*2 and CYP2C9*3 alleles confer a lower risk for myocardial infarction. Clin. Chem..

